# Navigating inherited “intensive surveillance” within Japan's structural norms of autonomy: maternal identity negotiation among long-term Peruvian migrant mothers (1990–2025)

**DOI:** 10.3389/fsoc.2026.1944754

**Published:** 2026-10-12

**Authors:** Katia Sara Huaman Palomino, Yoshitsugu Hirata

**Affiliations:** 1 Graduate School of Humanities and Social Sciences, Okayama University, Okayama, Japan; 2 Faculty of Education, Okayama University, Okayama, Japan

**Keywords:** acculturation, intensive surveillance, Japan, maternal identity, Peruvian migration, reflexive continuity, Third Space, transnational motherhood

## Abstract

**Introduction:**

This study examines how Peruvian mothers who have settled in Japan for decades rework their identities as mothers. These women were raised with the belief that being a good mother means constantly watching over and protecting their children. But in Japan, child-rearing pushes children toward independence from a young age. This study asks: What happens when these two different approaches of mothering meet?

**Methods:**

We interviewed 21 Peruvian mothers who had lived in Japan for 17–35 years.

**Results:**

The analysis identified four identity positions to this cultural friction. The first is holding firmly to Peruvian maternal scripts (“Ideological Retention”). The second is following Japanese conventions in public while keeping Peruvian ways at home (“Compartmentalization”). The third is moving away from inherited protective scripts and adopting Japanese norms of independence (“Assimilation”). The fourth is blending elements of both into a maternal self that is “neither the one nor the other” (a “Third Space”).

**Discussion:**

This study helps us understand that migrant mothers do not simply choose between their home and new cultures. Instead, they actively work to create a way of mothering through a process we call Reflexive Continuity that makes sense for their unique situations.

## 1 Introduction

Migration reshapes a person's life in far-reaching ways. It sets off a continual process of self-identification in which immigrants repeatedly revise how they see themselves along racial and ethnic lines. This process is shaped by both the receiving cultural context and their own choices (Chen and Li, 2025). When that immigrant is a mother, or a woman who takes up motherhood after arriving, the shift reaches past the individual. It reworks the dynamics and structure of her family (Tummala-Narra, 2004) and gives rise to new ways of functioning within the household and in the wider host society (Gonzalez and Ruiz-Casares, 2022).

Scholarship on migration has increasingly turned to immigrant mothers, treating motherhood as a primary site where ethnic and gendered subjectivities are continually negotiated (Cappellini and Yen, 2016; Zhu, 2020; Zou et al., 2025). For Peruvian migrants in Japan, this discussion is situated within the history of the Nikkei diaspora (descendants of Japanese emigrants commonly referred to in migration literature as Nikkeijin) and Japan's 1990 Revision of the Immigration Control and Refugee Recognition Act. This major reform created a legal pathway allowing second- and third-generation descendants of Japanese emigrants, together with eligible family members, to live and work in Japan (Takenaka, 2004). This policy contributed to increased migration from Latin America, including Brazil and Peru, during the 1990s.

Peruvian migration to Japan belongs to this wider pattern of Latin American mobility toward East Asia. Researchers have documented at length the labor trajectories and ethnic hierarchies of this Nikkei diaspora (Lagones, 2015; Sueyoshi, 2017; Takenaka, 2009; Takenaka and Paerregaard, 2015). Nonetheless, compared with the more thoroughly studied Brazilian Nikkei community, Peruvian migrants in Japan have drawn limited scholarly attention, even though they form a distinct group with particular characteristics (Takenaka and Paerregaard, 2015). They came to Japan through the legal pathway opened by the 1990 revision of the Immigration Control Act, at a moment when Peru was gripped by hyperinflation and by the political violence of the Shining Path insurgency. Together, these conditions generated powerful push–pull dynamics that carried thousands of Peruvians toward Japan (Takenaka, 2004, 2014). These macro-structural forces also meet individual maternal biographies. Furthermore, the contemporary Peruvian community has grown far more heterogeneous. It now includes Nikkei descendants of different generations as well as non-Nikkei migrants and family members scattered across industrial zones and more isolated regional prefectures.

“Intensive motherhood” has been theorized extensively in Western contexts as a gendered expectation. It requires mothers to devote enormous amounts of time, energy, and resources to raising children (Hays, 1996). Recent scholarship has extended this ideology by foregrounding how race and class shape its expression across different maternal populations (Minnotte, 2023). This expectation is supported in Japan through the cultural ideology of the *ryōsai kenbo* (good wife, wise mother) which has historically placed the primary contribution of women to society in the domestic sphere (Ezawa, 2016). For Peruvian mothers, such expectations meet their own cultural script of *marianismo*, a gendered ideal of female self-sacrifice and protective devotion that draws from Latin American Catholic tradition (Krotofil et al., 2026). Where these ideological frameworks overlap, the terrain for negotiating a maternal identity becomes curiously complicated.

Between 1990 and 2025 a visible but under-researched community of Peruvian migrants took shape in Japan. Among them, women who became mothers spent decades raising children while contending with a social environment that subjects mothering to a largely invisible yet pervasive form of intensive surveillance. This surveillance is not simply top-down monitoring by the state. It is embedded in what we term Japan's structural norms of autonomy: a framework in which maintaining a self-restraining subjectivity requires continual self-policing against appearing *wagamama* (selfish), and in which the *seken*, the surrounding social gaze, enforces conformity through informal sanction (Ito-Morales, 2022). Motherhood in this landscape has long carried a heavy charge of sacrificial devotion, with the ideal of the all-giving mother serving as an internal benchmark by which women measure their own performance and are measured by others (Ezawa, 2016).

A further pillar of Japanese maternal ideology is the *bosei shinwa*, the myth of maternal love and identity (Ao, 2015). This myth sets a demanding standard: a good mother is expected to subordinate her own needs, ambitions, and autonomy to the continuous, self-sacrificial care of her child. Alongside it runs the *mimamoru* approach, a strategy of “watching over.” Adults deliberately hold back from direct intervention and allow children to work through challenges on their own. At the same time, adults remain attentively present (Nakatsubo et al., 2021; Porter and Tanabe, 2023). Grounded in early childhood education, *mimamoru* is meant to cultivate children's autonomy and problem-solving. In domestic maternal practice, however, it depends on a high degree of maternal attentiveness: the mother must remain physically and emotionally present, constantly reading the child's state, while withholding overt help. Studies of Japanese mother–adolescent relationships confirm that maternal autonomy support operates within a relational frame. This frame prioritizes the child's integration into social networks rather than individualized self-expression (Sugimura, 2020). Contemporary research further shows how Japanese mothers' own cognitions about children and child-rearing shape their caregiving practices (Kawanishi and Doi, 2024).

What these norms imply for migrant mothers runs deep. When Peruvian mothers encounter the *bosei shinwa* and *mimamoru*, they are not merely observing unfamiliar parenting techniques; they are confronting a different philosophy of the mother–child bond. In the Peruvian frame, good mothering means physical presence, active protection, and emotional intensity. Matsumura (2019) describes it as the ideal of the mother to be a *gu*í*a* (guide), someone who maintains constant oversight, is closely involved in her children's daily lives, maintains a strong emotional connection with them, and helps them negotiate school and the wider social environment. A mother who does not walk her child to school, who lets her child sleep at a friend's house, or who does not know her child's whereabouts at every moment is understood to be falling short. In the Japanese frame, good mothering means trusting the child's developing competence, granting age-appropriate independence, and relying on the community's structural safety rather than on individual maternal vigilance. Japanese mothers have been found to grant young children discretion in areas such as play, clothing, and friendships as a way of encouraging autonomy and competence (Yamada, 2004). The *mimamoru* approach reflects a related practice: adults remain attentive but deliberately avoid unnecessary intervention so that children have opportunities to manage difficulties themselves (Kuramoto, 2022; Nakatsubo et al., 2021; Porter and Tanabe, 2023; Porter et al., 2025). These are not simply different preferences; they are competing moral frameworks about what a good mother is. Migrant mothers work through this conflict daily, both internally and in concrete decisions about the school commute, social activities, homework, and peer relationships. Each decision becomes an occasion for negotiating identity, as mothers weigh internalized Peruvian norms against the practical demands of raising children in Japan.

The Japanese institutional environment poses particular challenges. The school system in particular acts as a powerful site of cultural socialization that runs directly against Peruvian maternal values. Japanese schools expect children to walk to school on their own from the age of six, and to manage complex social hierarchies without parental intervention (Matsumura, 2019). Some of these situations involve *ijime* (bullying). For mothers who grew up where child abduction, street violence, and political instability were tangible threats, such expectations can feel deeply unsafe. The practice of *mimamoru*, developmentally appropriate in its own setting, can read as neglect from a Peruvian vantage point shaped by a very different risk environment. This institutional encounter between Peruvian protective scripts and Japanese pedagogical practice is not merely a cultural difference to be smoothed over; it is a fundamental challenge to maternal identity that demands active negotiation. It becomes a space where mothers confront the limits of their own cultural framework and develop new ways of understanding what keeping children safe means.

Our study therefore poses one overarching research question and three sub-questions. The general question is: How do long-term Peruvian migrant mothers in Japan reconfigure their maternal identity when their inherited cultural scripts of protection encounter the structural socialization and autonomy norms of the host society? The specific questions are: (1) How do long-term Peruvian migrant mothers in Japan mobilize inherited protective scripts, and to what extent do these practices function as cultural preservation or resistance? (2) How do past localized dangers shape these mothers' mistrust and risk perceptions in Japan? (3) How do long-term Peruvian migrant mothers use “intercultural mirroring” of Japanese mothering styles to redefine their maternal practices and ethnic identity?

To make sense of how these mothers move between the demands of Peruvian protective mothering and Japanese expectations of childhood autonomy, we draw on three bodies of work. The first is Berry's account of how migrants adjust to a new culture (Berry, 1997, 2003, 2005). Berry described four possible paths: fitting in fully (assimilation), holding to one's own culture alone (separation), balancing both (integration), or belonging to neither (marginalization). This scheme helps us see what mothers choose as they weigh cultural maintenance against societal participation. The second is Bhabha's notion of the Third Space (Bhabha, 1994), which captures the performative and agentic quality of these choices and describes how mothers hybridize cultural scripts into a new maternal self. Cultural encounters, for Bhabha, produce a liminal space of enunciation where identities are not simply chosen but mixed. This yields a state that is “neither the one nor the other” (p. 36), where a “foreign” or “untranslatable” element of the heritage culture persists as a trace that resists full absorption by the host society. The third move takes us beyond individual-level choices to how mothers are recognized and positioned by the host society. Following (Polynin, 2024) call to bridge acculturation psychology and empirical sociology, we situate these individual psychological processes within (Klarenbeek's, 2024) relational integration theory. This shifts the analytic focus from the individual's internal adaptation to how she is recognized by institutions, foregrounding the asymmetric power relations within Japanese social structures that often place a one-directional burden of change on migrant mothers.

The demographic reality of Japan's changing population lends this study particular weight. As Takenaka and Paerregaard (2015) explain, Peruvian migration peaked in 2007, when the Peruvian population reached roughly 60,000. Economic recession then prompted return migration, and official figures fell to 49,255 by 2012 (Ministry of Justice, 2025). As of June 2025, Japan recorded 49,220 registered Peruvian residents, of whom 23,594 are women Ministry of Foreign Affairs of Japan, 2025; Ministry of Justice, 2025. Although state data do not record their maternal status, they show that Peruvian women aged 15 or older number 20,492, or 86.85% of the female Peruvian population (Ministry of Justice, 2025). International frameworks define ages 15–49 as the standard range for reproductive and maternal potential. We follow this definition (United Nations, 2025; World Health Organization, 2026). Within this bracket, the primary cohort comprises 11,438 women. Another 9,054 women are aged 50–80 and over (Ministry of Justice, 2025). These groups are, demographically and socially, positioned within the life stage of family formation and intergenerational caregiving. Their relevance lies not in the numerical size, which can be considered small or large depending on the comparative frame (e.g., Japanese citizens, newer Asian or African migrant populations), but in their history of long-term settlement and decades of raising children within Japanese institutions. Understanding how they navigate the Japanese sociocultural terrain, we contend, calls for an intersectional lens attentive to gender, ethnicity, and migration status, as well as to the intergenerational transmission of maternal ideologies.

With one of the world's lowest birth rates and an aging society, Japan has come to rely on migrant labor to sustain its economy. The Peruvian Nikkei community, once imagined as a pool of temporary workers, has been a permanent feature of Japan's social landscape since the early 1990s and now includes 23,594 Peruvian women (Ministry of Justice, 2025). Japanese immigration policy nonetheless continues to operate under a fiction of temporariness. This raises a question about how migrant families, and mothers especially, build belonging and identity in a society that does not fully acknowledge their permanence. For migrant mothers, such precarity compounds the already demanding work of raising children in a foreign culture, children who are already, or are becoming, functional adult Japanese citizens.

Beyond describing these cultural shifts, we aim to contribute to migration theories of immigrant motherhood. Most studies describe how mothers change; we also ask why some things stay the same. The significance of this research lies less in the “what” and “how” than in the “why”: why do these mothers retain particular Peruvian values as markers of moral identity even after 35 years in Japan? This is why the date range (1990–2025) is explicitly included in the title of this manuscript. It captures the period across which participants' migration, maternal experiences, and identities developed and were retrospectively reconstructed. The year 1990 marks both the revision of Japan's Immigration Control Act, which opened a major legal pathway for Peruvian migration, and the earliest year of arrival among participants in our sample. The year 2025 marks the endpoint of the study, when the interviews were conducted and participants reflected on up to 35 years of residence in Japan. A common assumption holds that living in a new country produces a natural blending of cultures (Bhabha, 1994). Yet mothers, in our study, who have spent more than three decades in Japan show that change is neither simple nor linear. We introduce the idea of Reflexive Continuity to describe what actually happens: mothers neither absorb the new culture wholesale nor reject it outright. They think actively about what to keep from their Peruvian way of mothering and what to change. We argue that they experience cognitive dissonance as they observe host-society practice and interact with children raised in the Japanese system. That dissonance acts as a catalyst. It prompts mothers to compare, to evaluate, and then either to transform or to reaffirm their experiential capital and their Peruvian maternal scripts (the survival strategies and values cultivated in Peru and learned from their own mothers).

Given the cross-cultural nature of the study and participants' bilingual narratives, this study incorporates several specialized Japanese and Spanish concepts throughout the manuscript. We provide a consolidated glossary of core Japanese and Spanish terms, including their translations and contextual definitions in Table 1.

**Table 1:** Glossary of Japanese and Spanish terms.

Term	Literal translation	Contextual and cultural meaning in this study
Arubaito	Part-time job	A standard social practice (casual part-time employment) typical among Japanese students and homemakers in Japan; used by mothers to instill respect and independence
Bosei Shinwa	Myth of maternal love/instinct	The expectation that a good mother subordinates her own needs and autonomy to continuous, self-sacrificial childcare
Gomen nasai	I apologize/I'm sorry	A standard Japanese expression of apology, quoted as a school's repeated but unresolved response to a bullying complaint
Hoikuen	Nursery/daycare center	Early childcare facility for infants and toddlers in Japan, distinct from yōchien (kindergarten)
Ijime	Bullying	Peer harassment. Participants distinguish physical bullying from “mental” or “dirty” (psychological/social) bullying
Katazuke (“nai nai”)	Tidying up	Practice of organizing space and cleaning up after oneself, taught to Japanese children from infancy (colloquially prompted as “nai nai”)
Mamá gallina	Mother Hen	The study's central *in vivo* metaphor for the transnationally sustained protective/surveillant maternal identity
Marianismo	–	Gendered ideal of female self-sacrifice and protective devotion rooted in Latin American Catholic tradition. In this study, it anchors the intensive, highly protective maternal expectations
Mimamoru	To watch over/attentive waiting	Parenting approach of deliberate non-intervention, in which adults remain attentively present but let children work through challenges independently
Nanakorobi yaoki	Fall down seven times, get up eight	A traditional Japanese proverb symbolizing resilience and perseverance. In this study, it is invoked to describe a non-interventionist stance toward children's setbacks
Nikkeijin	Person of Japanese descent	Descendants of Japanese emigrants, distinguished from non-Nikkei Peruvian migrants in this study's sample
Nisei/sansei/Yonsei	2nd/3rd/4th generation	Generational classifications within the Nikkei diaspora, designating lineage distance from the original Japanese emigrants (Issei, or first generation)
Recelo	Mistrust/wariness	Theorized as transnational mistrust or structural skepticism that migrant mothers carry toward host-society institutions
Ryōsai kenbo	Good wife, wise mother	Cultural ideology locating women's primary societal contribution in the domestic sphere
Seken	Society/the world around one	Informal pressure: the public eye and community social gaze that enforces conformity through implicit sanction
Yōchien	Kindergarten	Preschool education facility for ages approximately 3–6

Definitions reflect conceptual usage within the participants' narratives and the authors' theoretical framing, not exhaustive lexicographic entries. Japanese terms are romanized using the modified Hepburn system with macrons indicating long vowels (e.g., *ryōsai kenbo*). Spanish terms retain standard orthography, including diacritics where applicable (e.g., *Mamá Gallina, marianismo, recelo*).

## 2 Materials and methods

### 2.1 Research design

This study adopted a qualitative paradigm and employed Reflexive Thematic Analysis (RTA). We followed the framework developed by Braun and Clarke (2006, 2019, 2021), which treats the researcher's subjectivity as an analytic resource and supports a culturally situated reading of meaning across participants' narratives.

### 2.2 Sampling and participant selection

Twenty-five potential participants were identified through snowball sampling during September and October 2025. The initial inclusion criteria were (1) Peruvian nationality or ethnic origin; (2) self-identification and experience as a mother; (3) residence in Japan; and (4) verbal agreement and written informed consent. A systematic screening procedure was then applied to retain only those who met the theoretical and demographic parameters. Four candidates were excluded on the predefined criteria set out in Table 2: one had not completed the informed consent form; two did not meet the criteria for maternal experience (non-mothers) or sufficient duration of residence in Japan; and one was removed on the study's gender criterion.

**Table 2:** Exclusion criteria for interview refinement.

Exclusion criteria	Participants (*n*)
Short-term residence in Japan (18 months) and not yet a mother	1
Resided >20 years but had no maternal experience	1
Male participant (Peruvian father)	1
Incomplete written consent documentation	1
Total participants excluded	4

The final sample comprised 21 Peruvian mothers who had lived in Japan for 17–35 years. Participants came from several prefectures, including Okayama, Hiroshima, Osaka, Hyogo, Gifu, Kagawa, Mie, and Yamaguchi. The sample included both Nikkei descendants (*Nisei, Sansei*, and *Yonsei* generations) and non-Nikkei participants. Ages ranged from 36 to 75 years, and educational backgrounds ranged from lower secondary to university degrees. Tables 3, 4 present the full demographic and migratory profiles.

**Table 3:** Migratory background context (*N* = 21).

ID	Nikkei generation	Residence in Peru	Migrant generation (age at arrival)	Year
P01	Non-Nikkei	Lima	1st Gen (27)	1990
P02	Yonsei (4th)	Lima	1.5 Gen (8)	1992
P03	Non-Nikkei	Ancash/Lima	1st Gen (33)	1992
P04	Sansei (3rd)	Trujillo	1st Gen (24)	1991
P05	Non-Nikkei	Trujillo	1st Gen (25)	1992
P06	Sansei (3rd)	Piura/Lima	1st Gen (39)	1991
P07	Nisei (2nd)	Callao	1st Gen (24)	1993
P08	Non-Nikkei	Arequipa	1st Gen (27)	1991
P09	Nisei (2nd)	Lima	1st Gen (23)	1990
P10	Sansei (3rd)	Callao	1st Gen (18)	1990
P11	Yonsei (4th)	Lima	1st Gen (22)	2008
P12	Sansei (3rd)	Lima	1st Gen (27)	2006
P13	Nisei (2nd)	Pisco/Lima	1st Gen (41)	1991
P14	Sansei (3rd)	Trujillo	1st Gen (33)	1998
P15	Non-Nikkei	Trujillo	1st Gen (23)	1994
P16	Non-Nikkei	Lima	1st Gen (23)	1993
P17	Non-Nikkei	Trujillo	1st Gen (26)	1992
P18	Sansei (3rd)	Piura/Arequipa	1st Gen (42)	1997
P19	Sansei (3rd)	Piura	1st Gen (44)	2000
P20	Sansei (3rd)	Lima	1.5 Gen (4)	1993
P21	Sansei (3rd)	Cuzco	1st Gen (23)	1993

Nikkei generation: 2nd (Nisei), 3rd (Sansei), 4th (Yonsei). non-Nikkei = individuals without Japanese ancestry. Migrant generation: 1st Gen (adult at arrival, age 18 or older); 1.5 Gen (minor at arrival).

**Table 4:** Current profile and adaptation in Japan up to 2025 (*N* = 21).

ID	Age	Education^a^	Japanese language^b^	Employment	Total children (born PE/JP)	Years in Japan^c^	Prefecture
P01	62	Short-cycle tertiary (ISCED 5)	3 Fluent	Full-time	2 (2/0)	35	Okayama
P02	41	Short-cycle tertiary (ISCED 5)	4 Native-like	Homemaker	2 (0/2)	33	Okayama
P03	66	Bachelor's (ISCED 6)	3 Fluent	Retired	3 (2/1)	33	Gifu
P04	58	Lower secondary (ISCED 2)	3 Fluent	Self-employed	3 (2/1)	34	Hiroshima
P05	58	Bachelor's (ISCED 6)	3 Fluent	Self-employed	2 (0/2)	33	Osaka
P06	73	Upper secondary (ISCED 3)	1 Basic	Part-time	3 (3/0)	34	Okayama
P07	56	Short-cycle tertiary (ISCED 5)	3 Fluent	Self-employed	3 (2/1)	25	Hiroshima
P08	61	Upper secondary (ISCED 3)	3 Fluent	Part-time	2 (0/2)	34	Hyogo
P09	58	Short-cycle tertiary (ISCED 5)	3 Fluent	Self-employed	3 (1/2)	35	Osaka
P10	53	Short-cycle tertiary (ISCED 5)	3 Fluent	Full-time	3 (0/3)	35	Mie
P11	39	Bachelor's (ISCED 6)	2 Fair	Full-time	1 (0/1)	17	Kagawa
P12	46	Bachelor's (ISCED 6)	3 Fluent	Self-employed	1 (0/1)	19	Hiroshima
P13	75	Bachelor's (ISCED 6)	2 Fair	Retired	4 (4/0)	34	Hiroshima
P14	60	Short-cycle tertiary (ISCED 5)	2 Fair	Full-time	3 (1/2)	27	Hiroshima
P15	54	Bachelor's (ISCED 6)	4 Native-like	Full-time	1 (0/1)	31	Hiroshima
P16	55	Upper secondary (ISCED 3)	3 Fluent	Part-time	3 (1/2)	32	Hiroshima
P17	59	Bachelor's (ISCED 6)	2 Fair	Part-time	2 (0/2)	17	Hiroshima
P18	70	Short-cycle tertiary (ISCED 5)	1 Basic	Part-time	2 (2/0)	28	Kagawa
P19	69	Lower secondary (ISCED 2)	1 Basic	Part-time	6 (6/0)	25	Kagawa
P20	36	Upper secondary (ISCED 3)	4 Native-like	Part-time	3 (0/3)	32	Hiroshima
P21	55	Upper secondary (ISCED 3)	3 Fluent	Self-employed	1 (1/0)	32	Yamaguchi

PE/JP = Peru/Japan. ^a^Education classified per the International Standard Classification of Education (UNESCO Institute for Statistics, 2011). ^b^Japanese proficiency self-rated: 1 basic = does not speak but understands a little; 2 fair = speaks a little and understands well; 3 fluent = speaks and understands well; 4 native-like = full fluency at a professional or native-like level. ^c^Participants returned to Peru temporarily (to visit children, reunite with spouses, for holidays or documentary procedures) without exceeding 5 years; for P07 and P17, extended stays in Peru lasted 7 and 16 years respectively and were excluded from their total years of residence.

### 2.3 Data collection and ethical considerations

Semi-structured interviews were conducted with the 21 participants in Peruvian Spanish. No interpreter was used as the first author, who conducted all the interviews, is a native Peruvian Spanish speaker. This direct linguistic communication allowed for cultural rapport and the authentic preservation of participants' narratives and metaphors. Furthermore, to achieve geographic diversity, we used Google Meet for 19 interviews and conducted two face-to-face interviews in Kagawa Prefecture with participants who had limited access to and comfort with digital tools. Ethical standards were maintained throughout: 17 participants provided written consent by postal mail, two electronically, and two in person. The study was conducted in accordance with the ethical guidelines of Okayama University and the Declaration of Helsinki. Table 5 summarizes the interview details.

**Table 5:** Data collection methods summary.

Parameter	Details
Primary platform	Google Meet
Face-to-face interviews	2 (Kagawa Prefecture)
Interview language	Spanish (Peruvian Spanish)
Informed consent	17 postal, 2 digital, 2 in person

### 2.4 Data analysis procedure

Reflexive Thematic Analysis followed the recursive six-phase framework of Braun and Clarke (2022): (1) familiarizing ourselves with the data and writing familiarization notes; (2) systematic line-by-line coding across the entire dataset; (3) generating initial themes through constant comparison and the clustering of codes; (4) developing and reviewing themes against the dataset to ensure internal coherence and external distinctiveness; (5) refining, defining, and naming themes; and (6) writing up with supporting quotations and theoretical integration. Data were managed in MAXQDA (version 26.2.1), which supported systematic coding, memo-writing, and theme development (see Figure 1 for the detailed research pipeline). All participant quotations in this manuscript are cited using the participant code followed by the MAXQDA paragraph position number (e.g., “P18, Pos. 21” denotes Participant 18, transcript paragraph position 21).

**Figure 1:**
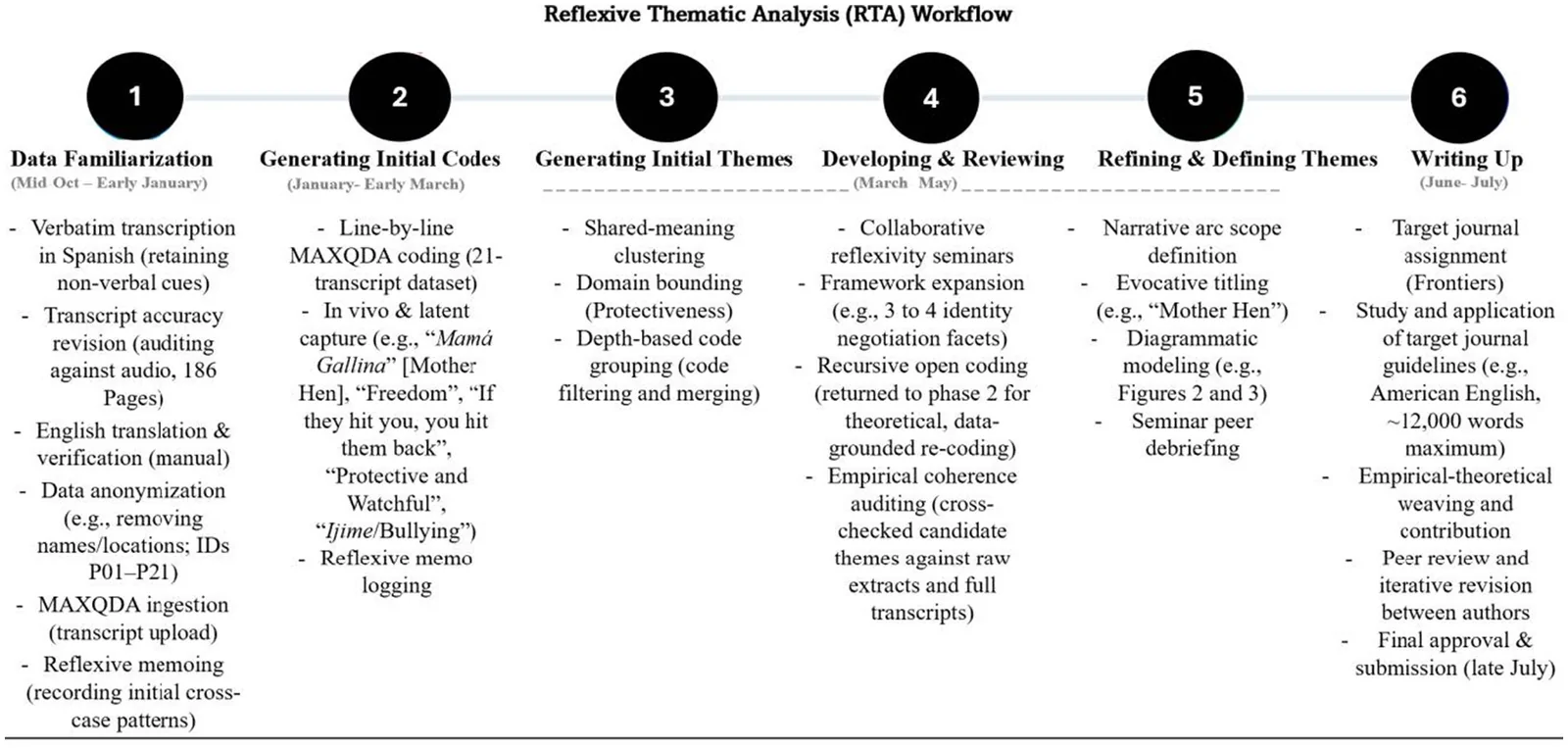
Six-phase qualitative research pipeline and reflexive thematic analysis workflow.

### 2.5 Positionality statement

In accordance with the Reflexive Thematic Analysis we adopted, we explicitly acknowledge our complementary positionalities as an active methodological tool to increase the rigor and credibility of this study. The first author's positionality as a Peruvian migrant Ph.D. student researching within her own community, generated rich, culturally grounded data that would have been difficult for an outsider to access. This underscores the value of community-embedded, insider research in migration studies, particularly for intimate domains such as mothering and family life. At the same time, the first author's academic training in a Japanese university provided the analytic distance needed for rigorous thematic interpretation. While this positioning also carries risks of over-identification and of assuming shared experiences, active reflexivity (RTA) and periodic meetings with the second author transformed this proximity into a methodological strength.

The second author is a Japanese male professor of education at a national university in Japan (Ph.D. in Education) specializing in the philosophy of education, and moral education (*dotoku kyoiku*). His positionality as an “outsider” to the Peruvian migrant community and as an “insider” to the Japanese societal institutional structures and parenting (e.g., *seken, mimamoru*, and *bosei shinwa*) provided a critical reflective counterweight to the analysis. The author's background created a space for reflective dialogue, especially during theme development (Phases 4 and 5). From this perspective, positionality in qualitative studies is not a limitation to be neutralized; rather, our complementary positioning served as a deliberate analytical tool to deepen reflexivity and ensure rigorous, culturally nuanced findings.

## 3 Results

Our findings are presented in two parts: demographic background and migratory patterns, followed by the reflexive thematic analysis. The thematic analysis is organized around three areas: (1) “I am a Mother Hen,” the transnational continuity of protection and surveillance as maternal identity; (2) socio-cultural dissonance and institutional encounters; and (3) identity negotiation and the emergence of the Third Space. As a conceptual map for the analysis that follows, Figure 2 traces the dynamic landscape: Peruvian mothers bring established cultural paradigms such as *Mamá Gallina* (protective surveillance), marianismo (self-sacrificial devotion), and reterritorialized fear (transnational hyper-vigilance). These undergo friction with Japanese expectations of child autonomy and *mimamoru* (watching over from a distance). Through a process of “Reflexive Continuity,” a conscious evaluation and re-authoring, mothers arrive at four primary coexisting identity positions: Ideological Retention (holding firmly to Peruvian scripts), Compartmentalization (following host norms in public while maintaining home scripts), Assimilation (adopting host autonomy norms), and Third Space (constructing a hybrid, “neither the one nor the other” maternal self).

**Figure 2:**
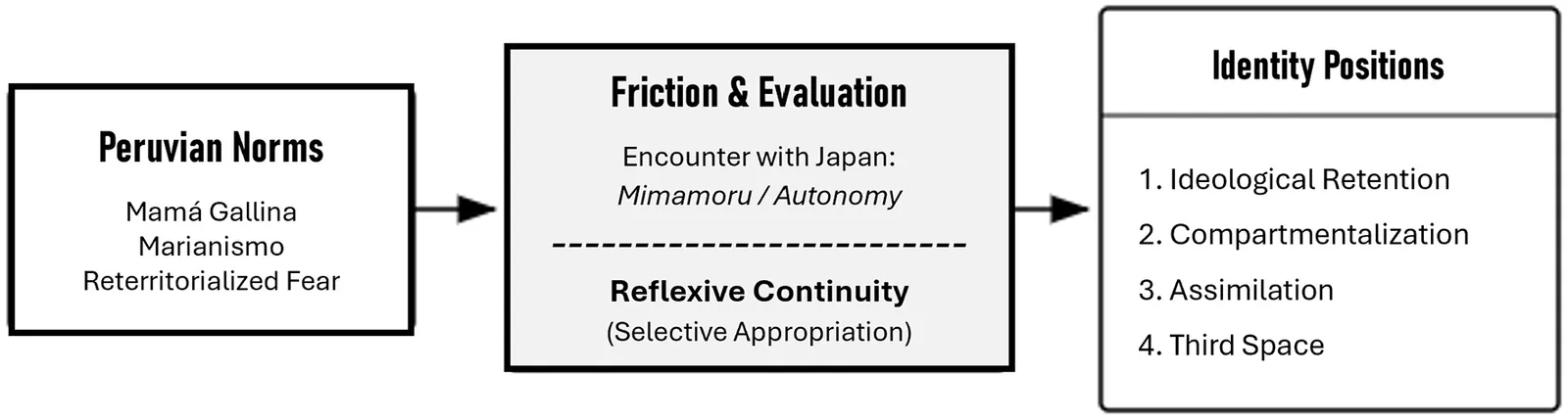
The maternal identity negotiation process.

### 3.1 Demographic background and migratory patterns

Most participants (76.2%, *n* = 16) migrated to Japan during Peru's socio-political and economic instability of the early 1990s, between 1990 and 1994, a period marked by hyperinflation and Shining Path violence. The latter refers to Peru's internal armed conflict, which began in 1980 when the Communist Party of Peru (Shining Path/Sendero Luminoso) launched an armed campaign against the Peruvian state. The conflict involved killings, bombings, intimidation, frequent blackouts, disappearances, and other forms of violence committed by insurgent groups as well as state security forces, producing widespread insecurity and displacement across the country.

These background conditions of living under a state of emergency, internal displacement among other social challenges in Peru, provide essential historical context for understanding their expressed *recelo* (mistrust) and protective (surveillant) maternal identity. They are also important for understanding the migratory profile of our participants, as they shaped a distinct demographic cohort of mothers fleeing not only financial but also physical precarity. P09, for example, stated directly that her main reason for coming to Japan was specifically to escape socio-political conflict and internal insecurity. She, being a Japanese descendant (*nikkeijin*), was able to enter the country under the 1990 amendment of Japan's Immigration Control and Refugee Recognition Act. Nikkei mothers and women, as well as non-Nikkei wives of Nikkei husbands, were eligible for Long-Term Resident or Spouse visas, granting them legal entry and unrestricted authorization to work. Married couples were able to enter together (e.g., P01, P13) or have one spouse follow the other after some time (e.g., P16, P17). Meanwhile, mothers with no direct Japanese ancestry (non-Nikkeijin who were not married to a *Nikkeijin*) entered by other means (e.g., purchasing Nikkei surnames, adoption), or occasionally overstaying their visas temporarily (e.g., P02).

Because this political violence and economic collapse affected both provincial regions of Peru (e.g., Trujillo, Ancash, Cusco) and the capital (Lima), our cohort reflects a broad demographic mix of Nikkei descendants, non-Nikkei spouses, and non-Nikkei single women across varied socio-economic backgrounds. *Nikkeijin* status was diverse, covering 66.7% (*n* = 14) of participants across the second (*Nisei*), third (*Sansei*), and fourth (*Yonsei*) generations. *Sansei* formed the largest subgroup (*n* = 9). The remaining 33.3% (*n* = 7) were non-Nikkei.

A “myth of temporality” shaped initial migration decisions: mothers framed their departure as a short-term move of 1 or 2 years, meant to accumulate capital before returning to Peru. As the stay lengthened, maternal guilt prompted reunification. P04 recalled the turn: “Thank God, because I worked well, the company was happy with my work, so I told them I wanted to bring my children” (P04tg7, Pos. 28). P07 expressed a similar resolve:

“I have to bring my daughters here, I don't care how it is … I decided to bring them” (P07, Pos. 67). These accounts show how the temporal extension of migration forced mothers to confront their maternal identity in a new cultural setting, and they explain the cohort's high average residence of nearly three decades (range 17–35 years).

At the time of interview (2025), the mothers ranged from 36 to 75 years of age, with an average of roughly 30 years of residence. Their profile skewed highly educated (66.7%, *n* = 14): Seven participants (33.3%) held a bachelor's degree or higher (ISCED 6), and seven (33.3%) held short-cycle tertiary degrees (ISCED 5). Most also reported strong Japanese proficiency, with 66.7% (*n* = 14) rating themselves “Fluent” or “Native-like.” The remaining participants gave several reasons for not learning Japanese. Factory work did not require it for P11, P13, P18, and P19. P06 also cited the expectation of a temporary return, while P17 had spent an extended period back in Peru. P14 had chosen to stay home and devote herself to child-rearing.

### 3.2 “I am a Mother Hen”: the transnational reconstruction of protection

For the mothers in this study, maternal identity is built around an uncompromising commitment to protection. Despite Japan's recognized safety, these women sustain a level of vigilance they voice through the *in vivo* metaphor of the “Mother Hen” (*Mamá Gallina*). The analysis shows that this protective identity is a transnational carry-over of learned practices and traumatic experience rooted in the Peruvian past, rather than a reaction to the host environment alone. The pattern recalls hooks's (1992) account of how marginalized communities develop oppositional forms of looking as survival strategies, although here the gaze is trained on child protection rather than resistance to domination.

#### 3.2.1 The intergenerational transmission of surveillance

The data indicate that protective behavior is grounded in an intergenerational inheritance in which good mothering is equated with close surveillance. Participants described childhoods in Peru governed by strict parental control. P01 traced her own disciplinary framework to her parents' strictness, recalling restricted mobility and early curfews: “I got that from my parents; they were very strict. My parents never let me go out after… I slept at 6 o'clock, I had to be home at 3 o'clock, I had dinner at 5 o'clock, I didn't go out at night” (P01, Pos. 44).

P16 used the image of a “butterfly in a glass case” to convey her level of maternal supervision. Though she remembered these measures as “suffocating,” she acknowledged replicating them with her own children in Japan, and she kept to them despite her husband's objections because they matched her internalized sense of moral duty:

My mother was very controlling with me, she took care of me as if I were a butterfly in a glass case … For example, when I was in high school, all my classmates would go to the museum, the theater, the beach, Chosica [a popular recreation town], or somewhere else; they would all go together, right? But my mom would say, “If you want to go, I'm coming too.” … I'm a person who, just as my mother took care of me, I've also taken care of my children. Even my husband used to tell me, “You protect them too much,” “You're protecting your son too.” He didn't like it. But … I did what I felt was right. The way my mother treated me sometimes seemed suffocating, but I understand her; my mother took care of me. (P16, Pos. 33–35, 48)

Surveillance here is not a passive instinct but a learned moral duty. These mothers reproduce the scripts in Japan to keep their children safe even where objective risks are markedly lower. P04 framed the protective ideal as a moral imperative to head off danger: “I always tell all moms: ‘Don't leave your children and the dangers that exist. We have to avoid them, we have to avoid them. I always tell them, ‘You have to avoid it' (P04, Pos. 125)”.

#### 3.2.2 Transnational hyper-vigilance and reterritorialized fear

Personal trauma and observation rooted in Peru reinforce the “Mother Hen” identity. P03, drawing on her professional background as an early-education teacher, described being “left with a bit of trauma” after witnessing parental neglect in Peru, which led her to forbid sleepovers and unsupervised socializing for her children in Japan (P03, Pos. 65). P10 echoed this: “And because of things that have happened to me in my life, I have never let them stay overnight at anyone else's house” (P10, Pos. 42).

P17's narrative shows how *recelo* (mistrust) becomes reterritorialized. An armed robbery in Peru functions not as a closed past event but as a “turning point” that stays cognitively active in Japan. The robbery hardened her distrust of public space and drove her definitive return to the perceived security of Japan with her adult daughters. Her fear and protective ideology persist despite Japan's safety because her internal risk assessment remains tied to the original trauma, sustaining a high state of alertness. Although her daughters are now 30 and 25, she identifies as a Mother Hen because she is “always looking out for them” (P17, Pos. 117).

This constitutes a reflexive bridge: the mother uses her experiential capital, the survival strategies learned in a high-risk environment, to evaluate host-society practice. Her vigilance responds not to local evidence of danger but to a preventive moral duty that crosses geography, casting the threat as universal rather than local. These accounts point to a transnational hyper-vigilance in which the mother's risk assessment stays tethered to the dangers of the home culture regardless of the receiving country's safety.

Transnational flows of information further sustain this protective identity. P08 shows how negative news from the country of origin can shape parenting choices in Japan. Despite initial interest in the Boy Scouts, she withheld her children after being informed of abuse within similar groups in Peru: “I heard so many things in Peru that I said, maybe out of fear, that they would join some group and there would be bad people there … That's why I've always preferred them to come to the house” (P08, Pos. 72). The Mother Hen does not rely on local evidence of danger alone; her vigilance is a preventive measure against a threat imagined as universal. Maintaining *recelo*, in this sense, is a strategic effort to protect the family against a world perceived as unpredictable.

#### 3.2.3 The case of 1.5-generation mothers

Our data analysis did not reveal a consistent Nikkei/non-Nikkei division in the protective maternal scripts identified in the interviews. Accounts of close surveillance, active protection, and the Mother Hen ideal occurred among both Nikkei and non-Nikkei mothers, and no stable progression was evident across Nisei, Sansei, and Yonsei participants. Nikkei background therefore did not function as a distinct organizing pattern for this particular domain of maternal identity. The clearest difference in the dataset instead concerned migrant generation and location of socialization: contrasting first-generation mothers with 1.5-generation mothers.

Set against the reterritorialized fear of most of the cohort, the narratives of the 1.5-generation participants, mothers whose childhood and education took place mainly in Japan, reveal trauma-informed protection grounded in personal experience of “mental” or “dirty” bullying suffered in Japan. These mothers fear that what happened to them will happen to their young children. “Mental and dirty” bullying names harm that is psychological rather than physical, as when, on the surface, “we're all fine, but then when the teachers leave, everyone ignores you, they act as if you don't exist” (P02, Pos. 57).

The only fear that I have and will always have is that my children will suffer bullying, because I have suffered it and I know that we are different, but bullying here, I don't know if you have heard of it, is a bit… It's quite dirty, so to speak. They don't hit you, they don't do anything to you, but mentally they attack you a lot, especially at the age of my children who are in primary and secondary school; it's very, very difficult, so I'm always afraid that my children are going to be attacked. I don't think they attack them because they are foreigners but for some reason; for example, my children are bigger than Japanese, their bodies are different, so I have the impression that one day they might say to them, “you have this big one, you have the other big one,” because they did it to me. That's the fear. (P02, Pos. 55)

P20, who arrived in Japan at age four and never returned to Peru, shared this experience. She too was bullied at school and described mixed feelings when her own children are teased:

Since I am a foreigner, my face is a bit different compared to Japanese people, and my way of speaking is also a bit different from other Japanese people. When my children's friends see me, if they are kind, they say, “Oh, your mom is different, she's a foreigner,” and they are happy. But there are also times when they say, “Eh… Your mom is a foreigner, so you are a foreigner too,” and they start to tease them. There are also annoying people who tease. (P20, Pos. 45)

Such stories circulate to mothers with younger children and lead them to distrust a Japanese educational system that, in their view, obliges them to fall back on inherited notions of hyper-vigilance and the Mother Hen identity. P12 recounted:

I didn't take my son to *hoikuen* nor *yōchien*. … I wasn't planning on sending him to [primary] school. … I thought, “I'm going to educate him myself,” “I'm going to educate him myself, and then I‘ll get him a Japanese teacher“ … I was very reluctant because I had read about *ijime* and all that stuff, so I didn't trust them, and everyone I know, their children suffered from *ijime*, everyone I know. … The Peruvian children I've seen attending Japanese schools, the little ones are more withdrawn. More withdrawn, more withdrawn. That's it, more withdrawn. They don't even communicate much with their own families, but there must be other reasons for that too; their parents are busy. But you notice when you see a child, for example a Peruvian child, and a child who has been educated in Japan, even if they are both Peruvian, there is a big difference. (P12, Pos. 67, 77, 90, 93)

She refused to send her son to *hoikuen* or *yōchien* because she “didn't trust the Japanese system” and did not want him to have a “Japanese brain” or “Japanese spirit” (P12, Pos. 89), which she associated with withdrawal and a loss of individuality. Her response is driven by a defensive, trauma-informed logic of risk mitigation.

A key distinction runs through the “Mother Hen” category. First-generation mothers perform Ideological Protection by replicating inherited Peruvian scripts, which strengthen in Japan; 1.5-generation mothers (e.g., P02, P20) engage in Trauma-Informed Protection. Their fears are not a cultural carry-over from Peru but a localized response to specific Japanese risks, namely the *ijime* (psychological bullying) they experienced during their own socialization in the Japanese school system. This fear extends to mothers now raising children of pre-school age. Hearing similar stories from peers, they come to see “protection” as multifaceted: it means safeguarding children from physical harm and from social exclusion alike.

### 3.3 Socio-cultural dissonance and institutional encounters

In analyzing how our participants navigate their maternal selves in Japan, we recognize that their experiences operate across two fundamentally distinct analytical levels: first, the perceived socio-cultural differences in inherited and encountered maternal scripts and how Peruvian mothers personally make sense of them; and second, the structural institutional policies, school environments, and systemic barriers that actively condition their maternal practices and challenge their maternal identity reconstruction. We examine these two levels separately.

#### 3.3.1 Dissonance with Japanese “freedom”

A primary catalyst for identity negotiation is the observation of Japanese mothering styles, which participants read as markedly more “detached” and autonomous, granting children “too much freedom.” This perception produces cultural dissonance, and the Peruvian mother comes to define her maternal identity partly by contrast with Japanese norms. Table 6 summarizes the core oppositions between the two paradigms that structure this dissonance, drawing together the ideological contrasts that the following accounts illustrate.

**Table 6:** Contrasting paradigms of maternal practice: Peruvian and Japanese norms.

Parenting attribute	Peruvian norm (*Mamá Gallina*)	Japanese norm (*mimamoru* and *ryosai kenbo*)
Primary focus	Close vigilance (intensive surveillance) and physical protection	Early childhood independence and autonomy
Maternal role	Active protection and intense emotional closeness	Watching over from a distance, allowing the child to learn from risk
Perception of danger	The outside world is full of risk, shaped by past trauma and reterritorialized fear	The environment (school and community) is relatively safe and supportive
Social expectation	The child is continuously supervised; leaving a child unattended is read as “neglect”	The child is encouraged toward age-appropriate independence, such as walking to school alone from around age six

The table condenses the ideological contrasts elaborated and evidenced across the results and discussion.

The protective framework of the Mother Hen shows up in demands for proactive protection. It stands against the perceived “freedom” of Japanese children because the participants' own standard for good mothering rests on constant communication and firm curfews:

Another difference [between Peruvian and Japanese mothering] is freedom … [In Japan] it is “I'm going out to play,” “Go ahead.” But when I told my mom, “I'm going to play,” she would say: “What time are you coming back?” “Where are you going?” She kept a close eye on me. (P20, Pos. 25)

Active surveillance extends to how children dress. P01 explained:

I have always raised them my way; they are men, my sons. I have never allowed them to dye their hair, like many mothers who accept earrings, too. Until now, my sons, well, they tie their laces well; they always wear them because they've got used to it; they've never had that… I mean, I haven't allowed them to wear earrings, to wear this or that new fashion. No, no, no, no, I've never allowed them to do that. I think that's quite different from the Japanese upbringing. They give a lot of freedom. (P01, Pos. 54)

The autonomy granted to Japanese children is read as a risk of moral laxity and exposure to social deviance, and also as a safety risk. P14 illustrated the contrast in protective practice when she was struck by the absence of maternal check-ins among her child's Japanese friends:

It shocked me at first, because here children are used to staying at their friends' houses … The first time all the children were here, I told my son, “Tell your friends to call their mothers to let them know they're okay.” They said, “No, it's okay.” So I said, “That's strange.” (P14, Pos. 52, 56)

Although Japan is recognized as safe, the mothers often read the independence, or “freedom,” afforded to Japanese children not as a developmental strength of the host culture but as a sharp departure from their own norms of maternal involvement. P10 characterized Japanese mothering as “more relaxed,” a script she consciously avoids in favor of the surveillance and strictness she inherited:

What I imitated from my mother is being a little strict with them, not being as relaxed as Japanese people are here, letting them go out at any time. They have times to come home, times to go out, to let me know where they are, what they are doing, what they are not doing … but they always have to tell me, “Mom, I'm here.” (P10, Pos. 42)

Surveillance for the sake of protection remains central to their identity; the Mother Hen is reconstructed in Japan as a cultural counterpoint. It is a state of being “always there, watching” (P11, Pos. 42) that provides security and identity in a society perceived as detached.

#### 3.3.2 Institutional barriers and responses to bullying

While socio-cultural dissonance manifests in daily domestic decisions, it is significantly compounded when mothers interact with formal host institutions like the Japanese school system. Here, exercising this protective surveillance runs up against the Japanese institutional, legal, and social system, and the collision produces a sense of “impotence.” P03 noted that while Peruvian scripts sanction direct parental intervention to protect a bullied child, “Japanese law doesn't allow adults to put themselves on the same level as teenagers”; mothers must “restrain” and “deprive” themselves of their protective instincts to avoid legal trouble (P03, Pos. 51).

The narratives also point to the failure of Japanese schools to intervene effectively. P17 described how her daughter returned home with “bruises on her legs” and her “hair undone,” while the school offered only a repetitive and hollow “*Gomen nasai*” (I'm sorry):

At first, everything was normal … Then I started to notice that she was coming home crying … and I would go and complain … I would take her with me, ask for time off work, and talk to the principal … I would say, “But how is this possible? Look, she comes with her face looking like she's been crying, with her hair and braids undone.” They pulled her braids, they did *ijime* to her. It was awful, miss, because it hurt me so much to see my daughter with her face looking like she had been crying and had dried her tears. Then she would come without her bows. I always sent her with her little bows … Then I would notice bruises on her legs, her foot, because the boy, the child, he would take a ball and throw it at her … I mentioned it to my husband, and the incidents didn't last for a month or two months, it was practically a long time. He said to me, “I'm not going to put up with this situation with [Name of daughter],” he said, “P17, you're going to Peru.” Because the bullying was every day. … I went to school, and the principal said, “Oh! *Gomen nasai, gomen nasai*, ma'am.” And it was always “*gomen nasai*” … I was going to complain, and the principal just said to me, “*gomen nasai*, ma'am, *gomen nasai*.” I said to him, “But talk to the boy's father so he can correct him. How can he keep hurting my daughter? This happens every day.” (P17, Pos. 42–44)

In Klarenbeek's (2024) terms, the school here acts as a “recognizer” that fails to grant the mother “standing as equals” by dismissing her concerns. This helps explain why P17 returned to Peru and the 16-year gap noted in Table 4. P14 also left Japan over a similar situation and returned a year later because her son “missed his dad” and promised her he would “be stronger,” would no longer let others bully him (*ya no me voy a dejar*), and would “overcome all of this” (P14, Pos. 35). P16 likewise pointed to institutional misrecognition: a teacher advised her bullied daughter to “just ignore them,” which placed the burden of change on the victim, who eventually “changed the way she ate” to end the harassment (P16, Pos. 90).

### 3.4 Identity negotiation and the “Third Space”

The tension between Peruvian protectiveness and the perceived detachment of Japanese mothering leads to a re-authoring of the maternal self. Mothers do not simply retain their original ideology (Ideological Retention). They may deliberately re-author through a balanced identity (Compartmentalization), assimilate into host practices (Assimilation), or re-author themselves into a hybrid identity (Third Space) that blends Peruvian devotion and Mother Hen protection with a strategic adoption of Japanese-influenced detachment and ideals of children's independence.

#### 3.4.1 The “softening” process: from rigidity to flexibility

This evolution is often described as a “softening.” P11 lets her daughter enter Japanese social spaces such as an *izakaya* (gastropub), provided she keeps a “watching eye” (P11, Pos. 42). P14 traced her shift from a rigid Peruvian mother who forbade her eldest child from visiting friends' houses to a more flexible approach with her younger children, as she recognized the need to accommodate their peers' social world:

I used to say they couldn't be away from home … With the second one, things changed quite a bit, because all his friends came to our house. So, my son would say to me, “But why do they come? Why can't we go to their house?” I told him, “It's because I'm a Peruvian mom, I'm not Japanese.” … Later, I changed my mind based according to the situation … It's not easy being a mom; sometimes we make mistakes as moms, but we try our best (P14, Pos. 52–53)

The self-categorization “I am a Peruvian mom” works as an initial defensive boundary (ideological retention) that later gives way as she recognizes the social necessity of her children's peer integration (compartmentalization): “But I would tell my son, ‘If you go to their house, you have to call me, because otherwise I'll get upset' ” (P14, Pos. 56).

Children act as a key catalyst for reconfiguring protective practices, especially when they grow up in Japan from an early age. This intergenerational dynamic helps explain why identity reconstruction is an ongoing, longitudinal process. Because Japanese institutions offer little targeted integration support or communicative agency to migrant families, and provide scant maternal cultural translation, opportunities to move away from strict ideological retention rarely arise early. The shift tends to come later in the life course through a kind of reverse intergenerational socialization, in which Japan-educated children and grandchildren act as cultural mediators who ease their mothers toward more balanced maternal spaces. P01 reflected:

The way my parents forced me. I also forced my children, “You have to eat to be strong.” Our upbringing in Peru, right? … Now, well, the older one doesn't complain. The younger one tells me, “Mom, you forced me to eat, and that's not done with children.” That's why sometimes he's afraid to leave my granddaughter with me because he thinks I'm going to force her. But that's not the way it is, either. I believe that there are two very important things: not to force, but there is also a limit that has to be seen, that if the child… the babies don't know, “I'm not hungry,” they say, to play, but they become malnourished, don't they? That's my way of thinking. (P01, Pos. 64, 66)

P01 contrasts Peruvian and Japanese realities. In Peru, she notes, children are valued for eating enough to grow “strong,” and so are made to finish their plate. Her reflection shows that Peruvian migrant mothers often do not decode internal Japanese social norms on their own; their understanding is filtered through the pushback and lived experience of their Japan-raised children. When deeper cultural comprehension stays out of reach, participants tend to default to ideological retention even while attempting behavioral assimilation under the pressure of demanding jobs. P06 is a case in point: she recognizes the practical necessity of Japanese autonomy because her daughter had to “take responsibility from a very young age” (P06, Pos. 80).

Her daughter managed her own schedule and bought her own obento (packed lunch) while P06 worked. Yet P06 still defines ideal motherhood through the lens of *marianismo*, holding that a good mother should be wholly “devoted” and “attentive.”

Where Japanese institutional agency does actively exist, the underlying *recelo* of Peruvian mothers begins to ease. P12's narrative shows this clearly. Despite her early reluctance to enroll her child in a Japanese school, she found herself “very surprised” by how supportive the teachers were:

I didn't expect that from the school, they are supporting me. They even told me, “*Okāsan*, if suddenly [son] gets sick, maybe he gets sick a lot because he hasn't gone to *hoikuen*. He's not used to school.” And they have supported me a lot. I think I'm privileged to be able to raise my son here in Japan. So yes, Japan does its best to support those of us who have children. (P12, Pos. 105)

In Klarenbeek's framework, integration is not a process “immigrants go through” but a relational change among all members. P12's softening of *recelo* occurs precisely because the school shifts from a rigid gatekeeper to an active recognizer. By letting her son bring a lunch box that validated his Peruvian eating habits (Pos. 79), permitting P12 to remain in class for 2 months (Pos. 89), and pursuing anti-*ijime* efforts (Pos. 91), the institution treated her concerns as socially valuable rather than negligible. These are structural attempts to ensure that being the “only foreigner in his class” (Pos. 91) does not translate into misrecognition or second-class status, acknowledging her and her son as equal members entitled to use resources in ways that respect their particular needs.

The relational equality the teachers established reduces the hierarchical distance typically found in Japanese institutions. This supports Klarenbeek's argument that integration problems are often institutional failures of recognition that can be addressed through pragmatic, egalitarian communication. The school's proactive support functions as a form of social legitimation, shifting the burden of “adaptation” away from the mother and child and onto the membership relationship itself. The result is a genuine desire to “learn everything” (Pos. 144) and to contribute: “I hope that my son, being the way he is, will contribute something” (Pos. 145); “I also hope to be able to talk to other moms too, right? That's it.” (P12, Pos. 107).

#### 3.4.2 Critique of overprotection and the embrace of strength

Several participants criticized the common Peruvian model of “overprotection” and intense attachment, linking it to the creation of dependent, “weak” children. P21 rejected what she called a “mama's boy” syndrome, arguing that her duty in Japan is to build in her son the “mental and emotional strength to face the trials of life” (P21, Pos. 45). She traced her learning about detachment to her study of Japanese philosophies:

I really love *reiki* [spiritual energy], a little bit of spirituality, that kind of thing. I love *Zen*. I love Japanese philosophy because it's really a philosophy of life. I've read a lot about these things, and I've more or less acquired that kind of mindset, right? (P21, Pos. 39)

P03 adopted a “pusher” rather than “protector” identity, citing the Japanese principle of *nanakorobi yaoki* (fall down seven times, get up eight):

The Peruvian mother [if the child] fell down, “Aaaaaay!”, you run, you pick him up … and we teach the child to be more dependent. I think this is overprotection … later, when they grow up, they are always reaching out to their mother. … The Japanese upbringing is, “if you fall down, you get up.” And if you fall down, “oh, bravo, good, you fell down, but you can, you can! Get up, get up! You can do it.” (P03, Pos. 45)

Working from this principle, P03 drew on Japanese parenting's emphasis on extracurricular activity to avoid the Peruvian habit of overprotection and to become a “pusher,” keeping her children busy to build their capacities: “They were always busy with something, whether it was sports… swimming lessons… dance… karate… kendo… volleyball… all kinds of sports, abacus” (P03, Pos. 47). Where P03 emphasized academic and physical strength, P09 valued moral strength and respect over “spoiling” (*engre*í*r*) her children. She used the Japanese custom of the part-time job (*arubaito*) to keep them from becoming arrogant or dependent, as she had seen in Peru:

Sometimes I see that mothers in Peru spoil their children so much that in the end they can't educate them … I didn't want to do that with my children … in Peru, [wealthy] children don't do anything anymore … they just want to boss around employees, yell at them, and even insult them. I never wanted to do that. … [My children], since they were in *kōkō* [secondary school], they did their *arubaito* … just like everyone else; they greet people, they are respectful, and that is what I have wanted to do since they were born here. (P09, Pos. 40)

The choices of P21, P03, and P09 illustrate assimilation through the reduction of cultural distinctions. By rejecting the Peruvian model of overprotection and taking up Japanese philosophies such as *nanakorobi yaoki*, Zen, *arubaito*, and extracurricular activity, these mothers work to shed “ethnic salience,” aligning with majority standards of autonomy in order to build their children's mental and moral strength and practical skills.

#### 3.4.3 Bhabha's Third Space

When neither ideological retention, nor compartmentalization, nor assimilation suffices, mothers inhabit Bhabha's (1994, p. 36) Third Space: a “neither the one nor the other” site where new maternal identities take shape and where blurred, untranslated elements from the country of origin persist. As P02 put it, “I don't like to identify with either side; I want to be myself, [name], I want to be me but always taking the good and beautiful things from both sides” (P02, Pos. 85).

She keeps her Peruvian “warm heart” and affection but fuses it with Japanese independence by making her children responsible for their own breakfast and tidiness. By refusing a “100% Peruvian” or “100% Japanese” binary, she constructs a hybrid self within this liminal space.

P05 embodies this hybrid state, validating two conflicting cultural scripts at once when she took her children to Peru:

I took them when they were very small and we had a meeting at home, at my parents' house … And then, the toys, the things, the snacks ran out… the ones who went to clean the house, in other words, were my children. The other children went home, and it was my children who had to tidy up, clean up, as they say “the *katazuke*.” They always tried to tidy up and clean. The Peruvian children left, they all left, they didn't tidy up. My children would say, “Mom, why don't they do that? Why don't they do that?” But my children did. They always, from the time they are very young, they are taught the “*nai nai*” (you have to tidy up). When they are babies, they just play with their toys, they tidy them up in their boxes, that is, they leave everything clean when they go to visit a friend. And we don't do that in Peru. In Peru we have a party, everyone goes away and leaves those things like that. My children ask me: “And why don't they tidy up, Mom?” They told me like this. So, I said, well, I kept quiet and just smiled, “because they are the guests”, that's all I said. But that's what guests do in Japan: they always leave you a clean house. (P05, Pos. 55)

While P05 admires the independence and responsibility of Japanese children in cleaning up after themselves, she refuses to pathologize the Peruvian custom as “wrong.” She holds both scripts in an ambivalent balance that resists full assimilation.

This hybrid negotiation extends to the meeting of inherited protective scripts and religious practice. For Peruvian mothers, as P07 observed, “God” stands as a symbol of protection and a source of unconditional help for the family's security (P07, Pos. 63). P02 takes this further into a Third Space negotiation. Rather than preserving institutional Catholicism intact, she translates and hybridizes her faith for her Japan-raised children:

For example, you know that in Peru we have the custom of baptism, communion and all those things. Look, I tell you, I believe in God, but I believe that God is not in the church, nor is he in the pope, nor the priest, nor those things; I believe that God is everywhere. So, I tell my children that all life on this earth must be respected, whether it is a spider, a pig, or an ant; one must not kill. And here in Japan they also care, they also believe that God exists in everything, in all things. So, that side and the side of Catholicism that we have in Peru, I have merged them, and I have taught my children that everyone's life is worth something. That is what I have taught my children: “don't kill even a little plant for fun, don't kill even a little pig.” I tell them, “We have to respect, and the weakest must always be protected.” That's what I tell my children: “a puppy, a kitten, we must never, never treat them badly; we must treat them well, because if we treat them well, then we will be better people.” So that is the fusion that I think I have made between Peru and Japan. (P02, Pos. 77)

Here P02 merges the moral framework of Peruvian Catholicism with the animistic, relational worldview of Japanese Shinto-Buddhist culture, in which *kami* or divinity resides in all things. By reframing Christian stewardship as a universal respect for life, she builds a protective moral anchor for her children.

Taken together, these narratives show that long exposure to Japanese society drives a marked evolution in maternal identity. The renegotiation is neither quick nor easy. Over time, some mothers come to see that certain stances create social difficulties for their children and begin to change. They do not abandon their values overnight but gradually adjust their practices in response to their children's social needs and their own growing familiarity with Japanese norms. This reconciliation is conditional and hard-won. It develops over years as mothers observe Japanese society's safety, learn the language, see their children demonstrate responsibility and well-being, and encounter Japanese institutional agency. Identity negotiation, then, is a spectrum: original norms are not discarded but reconfigured through a dialogue between deeply held inherited scripts and the host environment.

## 4 Discussion

This study asked how long-term Peruvian migrant mothers in Japan reconfigure their maternal identity when their inherited scripts of protection encounter the structural socialization and autonomy norms of Japan. Our findings show that they actively observe, compare, and evaluate inherited scripts against Japanese norms, a process we call Reflexive Continuity. When they watch Japanese mothering, participants do not absorb it unthinkingly. They notice differences, weigh those differences against their own values, and then decide what to keep and what to change. When P20 remarks that Japanese children have too much “freedom” while her mother “kept a close eye” on her, she is comparing. When P03 adopts *nanakorobi yaoki*, she is comparing and selecting a Japanese value that aligns with her own beliefs. Cultural learning here is not a one-way street on which migrants simply adopt the new culture; it is a transformative process of active choice and evaluation.

The first sub-question asked how mothers mobilize inherited protective scripts, and how far these practices function as cultural preservation or resistance. Here the narratives foreground the Mother Hen metaphor. Good mothering is equated with intensive surveillance and control, physical presence, direct defense (which broadens into safeguarding against physical harm and social exclusion), and religious practice. When mothers read Japanese autonomy as “neglect” or as “freedom” that courts moral laxity and risk, they hold to these scripts as a moral imperative to keep the family safe.

The Mother Hen metaphor is more than a descriptive label; it names a deep structure of maternal identity that is at once protective, transnational, and resistant. Drawing on (Haggerty and Ericson, 2000) concept of the “surveillant assemblage,” we argue that these mothers' emotional regimes are not bounded by national territory but are sustained transnationally through communication technology, memory, and the intergenerational transmission of protective scripts. This extends (Vertovec, 2004) analysis of cheap calls as the “social glue” of migrant transnationalism by showing how emotional surveillance operates as a parallel transnational social field. The mother–child relationship becomes a site where surveillance is not merely imposed but performed as an expression of maternal love and moral responsibility. The finding is consistent with (Orellana, 2009) account of the “para-ethnographic” cultural-translation work of immigrant families, though here the translation is maternal rather than linguistic.

Turning to the second sub-question, on how past localized dangers shape Peruvian migrant mothers' mistrust and risk perceptions in Japan, the data show that survival strategies learned in high-risk environments such as Peru (surveillance, direct defense, religion) are used to evaluate the host society. Fear becomes reterritorialized because internal risk assessment stays tethered to original traumas, whether social insecurity in Peru or bullying and social exclusion in Japan.

Our analysis reveals a critical distinction within the Mother Hen typology. First-generation mothers engage in Ideological Protection by reproducing the strict, intergenerational scripts inherited from their parents in Peru. In contrast, 1.5-generation mothers such as P02 and P20 perform Trauma-Informed Protection. For the younger group, surveillance is not a cultural carry-over but a direct response to Japanese risks met during their own socialization, chiefly psychological exclusion through bullying. Whereas, the first generation's fear is reterritorialized from Peru and broadens into protecting children from any harm or bullying in Japan, the 1.5 generation's protective identity is localized and structural: they fear the specific social exclusion they themselves suffered within the Japanese educational system. Having heard or read similar stories from compatriots, this fear reaches other young mothers whose children are not yet of school age.

Crucially, the absence of a stable Nikkei/non-Nikkei pattern in these protective scripts does not imply that Japanese ancestry is irrelevant to migrant maternal identity. Rather, it cautions against treating ancestry itself as evidence of cultural familiarity. The theoretical possibility that Japanese-descended Peruvian mothers might reactivate or reinterpret inherited affiliations after migration is important; however, we found little evidence that Japanese ancestry conferred distinct knowledge or cultural advantages regarding mothering in Japan. Instead, Nikkei mothers (*Nisei, Sansei, Yonsei*) consistently identified primarily as Peruvian mothers rather than Nikkei. Much like the non-Nikkei cohort, as their length of residence increases and daily interactions (and accompanying cultural dissonance) unfold, these women negotiate their maternal identities according to their individual realities. Thus, within the available data, protective scripts appeared across ancestral lines in both groups. As demonstrated above, the location of participants' formative socialization (first-generation vs. 1.5-generation) remained the primary axis of divergence.

The third sub-question asked how mothers use “intercultural mirroring” of Japanese mothering styles to redefine their practices and ethnic identity. The findings show that this mirroring drives identity negotiation. Through Reflexive Continuity, the negotiation yields four distinct identity positions: Ideological Retention, Compartmentalization, Assimilation, and the creation of a Third Space. Peruvian mothers in Japan do not all change in the same way; we observed four different paths. Some hold firmly to Peruvian mothering as a form of resistance (Ideological Retention); these mothers described traumatic experiences and strict parental control in Peru that they then replicated with their own children in Japan. Some changed their behavior in public settings, such as visits to a friend's house, while keeping Peruvian ways internally (Compartmentalization). Some deliberately assimilated to the receiving country (Assimilation); this group generally offered more detailed accounts of Japanese culture and mothering and expressed a more deliberate rejection of Peruvian overprotectiveness. Others created a new, hybrid mothering that translates the Mother Hen's protective devotion into the independence Japanese society expects (Third Space).

Our four types partly map onto Berry's well-known categories (Berry, 1997, 2003, 2005), but with important differences. Ideological Retention resembles Berry's “separation,” yet it is not merely holding to the old ways out of habit. These mothers choose this path because they believe their mode of protecting children is morally right. They interpret Japanese independence not as good parenting but as neglect. Compartmentalization resembles “integration,” where participants accept a balance of both cultures, acting Japanese in public while holding Peruvian values firmly within. Assimilation aligns with Berry's model because participants reject inherited maternal ideologies. These mothers criticize their peers' overprotectiveness and create psychological distance from their culture's maternal tendencies. Third Space also resembles “integration,” but it is more agentic and generative, through Reflexive Continuity, than a situational mixing of two cultures. Mothers who achieve it create wholly new ways of mothering that existed neither in their own maternal culture nor in the receiving one. Bhabha (1994) calls this the Third Space, a place where something genuinely new emerges. We found no evidence of Berry's “marginalization.” Figure 3 illustrates the multidimensional spiral of identity negotiation. Rather than mapping onto static quadrants, the model captures the dynamic interplay between the reflexive retention of Peruvian protection and the strategic engagement with Japanese autonomy-promoting practices.

**Figure 3:**
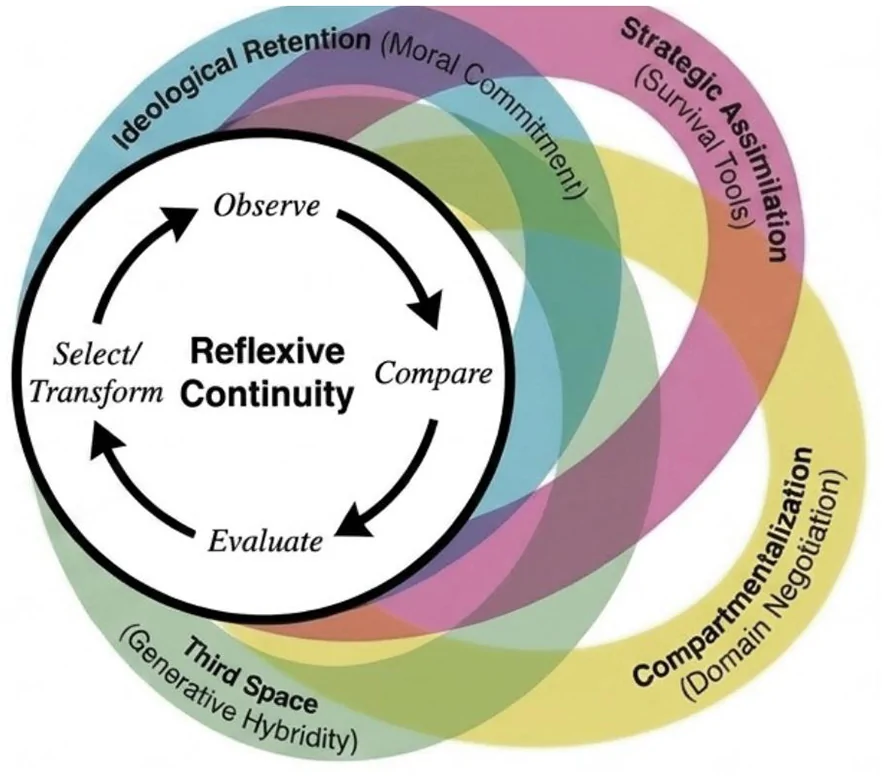
A spiral of maternal identity negotiation with the four identity positions.

Our findings thus extend Berry's multidimensional acculturation framework (Berry, 1997, 2003, 2005) by showing that maternal identity negotiation is not a static movement toward a fixed category but also a performative act of enunciation. Berry's typology identifies adaptive outcomes but often misses the Reflexive Continuity inherent in maternal work, where identity is not simply balanced but actively reconstructed and hybridized in (Bhabha, 1994) Third Space. Peruvian mothers reflectively observe and compare host norms with inherited scripts. They weigh the Mother Hen instinct for protection against Japanese expectations of childhood autonomy. From this reflexivity, they fashion a maternal self that is “quite literally neither the one nor the other” (Bhabha, 1994, p. 36).

Transnational ties and intergenerational friction (e.g., P02, P05) also persist, turning the “stubborn chunks” of heritage culture into resistant traces within the host society.

Building on Polynin's (2024) call to treat integration as a renegotiation of collective social identities, this study centers maternal agency and subjective interiority within the asymmetric power relations of Klarenbeek's (2024) relational membership framework. Schools, social workers, and support organizations in Japan should recognize that Peruvian mothers' protective behavior is not a problem to be fixed. It grows out of love and out of life experience in a more dangerous environment. Language matters here in particular: mothers who speak Japanese well are better placed to understand Japanese parenting norms, communicate with schools, and make informed choices about which practices to adopt. Schools should reach out to migrant mothers in ways that respect their cultural background rather than seeking to replace it. This principle of culturally respectful care is already established in healthcare (Attanasio and Hardeman, 2019; Bohren et al., 2015; Vedam et al., 2019), and our study shows it applies to mothering and identity as well.

The findings also speak to broader debates about gender, migration, and social reproduction. The work of mothering, such as feeding, clothing, educating, and protecting, is at once intimate and social. It is performed in private households but is shaped by the asymmetric power relations of host institutions, including public policy, cultural norms, and schools. For migrant mothers this reproductive labor takes on added dimensions. Beyond the practical tasks of childcare, they carry the emotional labor of cultural translation and the cognitive work of evaluating and comparing cultural norms. This work involves negotiating membership relations in a society where they may face institutional misrecognition. On top of this sits the identity work of reconstructing what it means to be a good mother across cultural boundaries. Maternal identity reconstruction, then, is not simply a psychological process but a form of social reproductive labor essential to a long-term integration in which everyone can enjoy standing as equals. The mothers in our study are not passive recipients of cultural change; they are active agents who produce new cultural meanings and practices at the intersection of Peruvian and Japanese maternal traditions. In doing so, they perform a crucial but often invisible role in shaping the next generation's cultural identities and social integration (Domingo, 2026). Recognizing this invisible work, maternal identity work as social reproductive labor, carries important implications for how receiving societies value and support migrant mothers. In line with Polynin (2024) and Klarenbeek's (2024), we contend that the task is to move away from civic-integrationist models that place a one-directional burden of change on migrant mothers. A better framework would value the renegotiation of collective social maternal identities within Japan.

## 5 Conclusion

This study shows that long-term Peruvian migrant mothers in Japan reconfigure their maternal identity through four distinct identity positions: ideological retention, compartmentalization, assimilation, and Third Space. Their reconstruction of identity is complex and many-sided. It cannot be reduced to straightforward assimilation or acculturation. The four identity positions are not rungs on a ladder of adaptation but distinct forms of agency, each with its own logic, strengths, and limits. Nor does the negotiation follow a single, linear path toward hybridity. It is better understood as Reflexive Continuity: a mode of work through which mothers meet cultural dissonance by observing, comparing across cultures, evaluating, and selectively taking up what serves them.

The Mother Hen metaphor captures a transnational protective identity sustained by intergenerational scripts and reterritorialized fears. First-generation mothers engage in cultural-ideological protection, reinforced by schools' failure to resolve bullying, while 1.5-generation mothers engage in Trauma-Informed Protection against localized Japanese risks such as *ijime*. Our findings extend (Kita et al., 2015) account of social isolation, language barriers, and cultural adjustment difficulties as key determinants of poor mental health outcomes among perinatal immigrant mothers in Japan. By bridging empirical sociology and psychology, migration studies can move beyond passive-adaptation models and better grasp the egalitarian struggle for standing as equals. Centering Peruvian mothers' voices contributes to a more nuanced understanding of migration, motherhood, and cultural change in contemporary Japan. It also reveals the resourcefulness and resilience with which migrant women raise children across cultures and build maternal identities suited to their circumstances.

The implications reach across policy and practice. For Japanese educational institutions, migrant mothers' apparent overprotectiveness should not be pathologized or dismissed. Our findings suggest that it should instead be understood as a reasoned response to intergenerational trauma and transnational risk perception. School outreach that addresses migrant mothers' concerns directly, offers transparent information about safety, and creates opportunities for intercultural dialogue between Peruvian and Japanese parents may build more trusting relationships. For immigration policy, the “myth of temporality” that influenced initial migration decisions continues to shape maternal identity decades later. Policies should acknowledge the permanent settlement of long-term migrants and provide pathways to secure residency, citizenship, and social integration. Such measures could reduce the precarity that sustains reterritorialized fear.

As global migration continues to reshape families and communities, the experiences of Peruvian mothers in Japan carry insights that reach well beyond this particular case. Their accounts show that mothering after migration is not simply a matter of adapting to a new culture. It means re-imagining what a good mother is when the old rules no longer hold and the new ones still feel foreign. The four identity positions we identified (holding on, balancing both, assimilating, and building something new) are not only ways of mothering; they are ways of being a migrant woman in the world. The Mother Hen who never stops watching, the performer who switches between cultures, the mother who lets go of her inherited scripts, and the hybrid mother who forges a third way all face the same problem: how to love and protect one's children while raising them in a world that is not one's own. Their creativity and resilience in meeting that challenge hold lessons for researchers, policymakers, and communities working to support migrant families in an interconnected world.

### 5.1 Limitations and future research

Several limitations warrant acknowledgment. Although the sample was geographically diverse across several prefectures, it relied on snowball sampling through ethnic networks, which may bias toward more connected individuals. Retrospective interviews covering 17–35 years of migration experience carry a risk of recall bias, particularly regarding early migration decisions and emotional states. The cross-sectional design limits our capacity to trace the longitudinal trajectory of identity change. Future research should use longitudinal designs to track maternal identity over time. It should also include fathers' and children's perspectives on maternal protective practices and compare Peruvian mothers with other Latin American migrant groups in Japan. Comparative work with Brazilian Nikkei mothers, who form a larger community in Japan, would be especially valuable.

Finally, our findings invite future scholarship to examine how distinct inherited maternal ideologies among Peruvian mothers in Japan clash with different dimensions of Japanese mothering, further illuminating how identity is negotiated under those conditions. For migration and family researchers, the study opens new questions about how Peruvian maternal identity negotiation operates in other domains of migrant family life, such as fathering, partnering, and grandparenting, and how these defensive scripts adapt, resist, and evolve across subsequent generations.

## Data Availability

The raw data supporting the conclusions of this article will be made available by the authors, without undue reservation.

## References

[B1] Ao A. (2015). Concepts of “motherhood” in Japanese literature: a discussion contributing to future education. J. Humanist. Educ. 3, 1–14.

[B2] Attanasio L. B. Hardeman R. R. (2019). Declined care and discrimination during the childbirth hospitalization. Soc. Sci. Med. 232, 270–277. doi: 10.1016/j.socscimed.2019.05.008 31112918

[B3] Berry J. W. (1997). Immigration, acculturation, and adaptation. Appl. Psychol. 46, 5–34. doi: 10.1111/j.1464-0597.1997.tb01087.x

[B4] Berry J. W. (2003). “Conceptual approaches to acculturation,” in Acculturation: Advances in Theory, Measurement, and Applied Research, eds. K. M. Chun, P. B. Organista, and G. Marín (Washington, DC: American Psychological Association), 17–37. doi: 10.1037/10472-004

[B5] Berry J. W. (2005). Acculturation: living successfully in two cultures. Int. J. Intercult. Relat. 29, 697–712. doi: 10.1016/j.ijintrel.2005.07.013

[B6] Bhabha H. K. (1994). The Location of Culture (London: Routledge).

[B7] Bohren M. A. Vogel J. P. Hunter E. C. Lutsiv O. Makh S. K. Souza J. P. (2015). The mistreatment of women during childbirth in health facilities globally: a mixed-methods systematic review. PLoS Med. 12:e1001847. doi: 10.1371/journal.pmed.1001847 26126110 PMC4488322

[B8] Braun V. Clarke V. (2006). Using thematic analysis in psychology. Qual. Res. Psychol. 3, 77–101. doi: 10.1191/1478088706qp063oa

[B9] Braun V. Clarke V. (2019). Reflecting on reflexive thematic analysis. Qual. Res. Sport Exerc. Health 11, 589–597. doi: 10.1080/2159676X.2019.1628806

[B10] Braun V. Clarke V. (2021). One size fits all? What counts as quality practice in (reflexive) thematic analysis? Qual. Res. Psychol. 18, 328–352. doi: 10.1080/14780887.2020.1769238

[B11] Braun V. Clarke V. (2022). Thematic Analysis: A Practical Guide (London: SAGE Publications). doi: 10.53841/bpsqmip.2022.1.33.46

[B12] Cappellini B. Yen D. A. (2016). A space of one's own: spatial and identity liminality in an online community of mothers. J. Mark. Manag. 32, 1260–1283. doi: 10.1080/0267257X.2016.1156725

[B13] Chen I. Li J.-C. A. (2025). Changing self-identification among immigrants in the United States. Front. Sociol. 10:1445287. doi: 10.3389/fsoc.2025.1445287 40415964 PMC12098439

[B14] Domingo P. (2026). Family dynamics in Filipino transnational families: the role of non-migrant mothers. Front. Sociol. 11:1752009. doi: 10.3389/fsoc.2026.1752009 41988102 PMC13076126

[B15] Ezawa A. (2016). Single Mothers in Contemporary Japan: Motherhood, Class, and Reproductive Practice (Lanham, MD: Lexington Books). doi: 10.5040/9781978731516

[B16] Gonzalez E. Ruiz-Casares M. (2022). Negotiating child agency in childcare practices among immigrant families. Child Fam. Soc. Work 27, 195–205. doi: 10.1111/cfs.12869

[B17] Haggerty K. D. Ericson R. V. (2000). The surveillant assemblage. Br. J. Sociol. 51, 605–622. doi: 10.1080/00071310020015280 11140886

[B18] Hays S. (1996). The Cultural Contradictions of Motherhood (New Haven, CT: Yale University Press).

[B19] hooks b. (1992). Black Looks: Race and Representation (Boston, MA: South End Press).

[B20] Ito-Morales K. (2022). Individual rights vs. common good? Asian Stud. 10, 69–95. doi: 10.4312/as.2022.10.1.69-95

[B21] Kawanishi C. Doi J. I. (2024). The influence of Japanese mothers' victim cognition regarding children and child-rearing on inappropriate child-rearing behavior. Curr. Psychol. 44, 275–287. doi: 10.1007/s12144-024-07113-y

[B22] Kita S. Minatani M. Hikita N. Matsuzaki M. Shiraishi M. Haruna M. (2015). A systematic review of the physical, mental, social, and economic problems of immigrant women in the Perinatal Period in Japan. J. Immigr. Minor. Health 17, 1863–1881. doi: 10.1007/s10903-015-0192-2 25784144

[B23] Klarenbeek L. M. (2024). Relational integration: from integrating migrants to integrating social relations. J. Ethn. Migr. Stud. 50, 233–256. doi: 10.1080/1369183X.2023.2259038

[B24] Krotofil J. Wo'jciak D. Metel D. (2026). Negotiating ideals: catholicism, intensive mothering ideology and gender norms in contemporary Poland. Eur. J. Women's Stud. 33, 138–152. doi: 10.1177/13505068261418795

[B25] Kuramoto M. (2022). Transitions in parents' perspectives and roles in children's cultural identity development: interviews with intercultural parents living in Japan. Educ. Psychol. Res. 70, 146–162. doi: 10.5926/jjep.70.146

[B26] Lagones J. (2015). Challenges of Nikkei Peruvian second generation in Japan: an overview of their employment status after the Lehman Shock 2008. Asian Soc. Sci. 11, 267–280. doi: 10.5539/ass.v11n26p267

[B27] Matsumura A. (2019). “Children here go to school alone.” motherhood at the crossroads of Peruvian migration in Aichi, Japan. Anthropologica 37, 85–106. doi: 10.18800/anthropologica.201902.004

[B28] Ministry of Foreign Affairs of Japan (2025). Peru basic data. MOFA. Available online at: https://www.mofa.go.jp/mofaj/area/peru/data.html (Accessed September 28, 2026).

[B29] Ministry of Justice Immigration Services Agency. (2025). Foreign residents by nationality/region, age (5-year age groups), and gender (table 25-06-03-1). Statistics on foreign residents in Japan (June 2025). e-Stat portal. Available online at: https://www.e-stat.go.jp/ (Accessed July 10, 2026).

[B30] Minnotte K. L. (2023). Decentering intensive mothering: more fully accounting for race and class in motherhood norms. Sociol. Compass 17:e13095. doi: 10.1111/soc4.13095

[B31] Nakatsubo F. Ueda H. Kayama M. (2021). Why don't Japanese early childhood educators intervene in children's physical fights? Some characteristics of the mimamoru approach. Early Child. Educ. J. 50, 627–637. doi: 10.1007/s10643-021-01184-3

[B32] Orellana M. F. (2009). Translating Childhoods: Immigrant Youth, Language, and Culture (New Brunswick, NJ: Rutgers University Press).

[B33] Polynin I. (2024). Relational integration/assimilation? A critical dialog with postcolonial and mainstream perspectives. Front. Polit. Sci. 6:1493637. doi: 10.3389/fpos.2024.1493637

[B34] Porter N. Tanabe K. (2023). The Mimamoru approach in contemporary Japanese parenting magazines: strategies for disciplining young children. Early Child. Educ. J. 51, 1241–1252. doi: 10.1007/s10643-022-01370-x

[B35] Porter N. Loveland K. A. Honda H. Yamane T. (2025). What is a good mother of children with autism? A cross-cultural comparison between the U.S. and Japan. J. Autism Dev. Disord. 55, 739–751. doi: 10.1007/s10803-023-06232-y 38265574

[B36] Sueyoshi A. (2017). Intergenerational circular migration and differences in identity building of Nikkei Peruvians. Contemp. Jpn. 29, 230–245. doi: 10.1080/18692729.2017.1351047

[B37] Sugimura K. (2020). Adolescent identity development in Japan. Child Dev. Perspect. 14, 71–77. doi: 10.1111/cdep.12359

[B38] Takenaka A. (2004). The Japanese in Peru: history of immigration, settlement, and racialization. Lat. Am. Perspect. 31, 77–98. doi: 10.1177/0094582X04264745

[B39] Takenaka A. (2009). “How ethnic minorities experience social mobility in Japan: an ethnographic study of Peruvian migrants,” in Social Class in Contemporary Japan: Structures, Sorting and Strategies, eds. H. Ishida, and D. Slater (London: Routledge), 221–237.

[B40] Takenaka A. (2014). The rise and fall of diasporic bonds in Japanese-Peruvian “return” migration. Int. Migr. 52, 100–112. doi: 10.1111/imig.12147

[B41] Takenaka A. Paerregaard K. (2015). How contexts of reception matter: comparing Peruvian migrants' economic trajectories in Japan and the US. Int. Migr. 53, 236–249. doi: 10.1111/imig.12001

[B42] Tummala-Narra P. (2004). Mothering in a foreign land. Am. J. Psychoanal. 64, 167–182. doi: 10.1023/B:TAJP.0000027271.27008.60 15138386

[B43] UNESCO Institute for Statistics (2011). International standard classification of education: ISCED 2011. Technical Report, UIS, Montreal.

[B44] United Nations Department of Economic and Social Affairs, Population Division. (2025). World fertility 2024. Available online at: https://www.un.org/development/desa/pd/ (Accessed September 29, 2026).

[B45] Vedam S. Stoll K. Taiwo T. K. Rubashkin N. Cheyney M. (2019). The giving voice to mothers study: inequity and mistreatment during pregnancy and childbirth in the United States. Reprod. Health 16:77. doi: 10.1186/s12978-019-0729-2 31182118 PMC6558766

[B46] Vertovec S. (2004). Cheap calls: the social glue of migrant transnationalism. Glob. Netw. 4, 219–224. doi: 10.1111/j.1471-0374.2004.00088.x

[B47] World Health Organization (2026). Women of reproductive age (15–49 years) population (thousands). Global health observatory indicator metadata registry. Available online at: https://www.who.int/data/gho/indicator-metadata-registry/imr-details/women-of-reproductive-age-(15-49-years)-population-(thousands) (Accessed September 29, 2026).

[B48] Yamada H. (2004). Japanese mothers' views of young children's areas of personal discretion. Child Dev. 75, 164–179. doi: 10.1111/j.1467-8624.2004.00661.x 15015682

[B49] Zhu Y. (2020). Transnationalizing lifelong learning: taking the standpoint of Chinese immigrant mothers. Glob. Soc. Educ. 18, 406–419. doi: 10.1080/14767724.2020.1762168

[B50] Zou M. Ouyang Y. Gao Q. (2025). Mothering in motion: migrant mothers' spatial negotiation of motherhood in urban China. Soc. Sci. 14:713. doi: 10.3390/socsci14120713

